# ENHANCING UPPER EXTREMITY MUSCLE STRENGTH IN INDIVIDUALS WITH SPINAL CORD INJURY USING LOW-INTENSITY BLOOD FLOW RESTRICTION EXERCISE

**DOI:** 10.2340/jrm.v56.40608

**Published:** 2024-09-24

**Authors:** Babak SHADGAN, Mehdi NOURIZADEH, Yekta SAREMI, Leila BAKTASH, Sepideh MORTEZANEZHAD, Stefan LAZAREVIC

**Affiliations:** 1 International Collaboration on Repair Discoveries, Vancouver; 2 Department of Orthopaedics, University of British Columbia, Vancouver; 3 Department of Pathology & Laboratory Medicine, University of British Columbia, Vancouver; 4 School of Biomedical Engineering, University of British Columbia, Vancouver, Canada

**Keywords:** spinal cord injury, exercise, blood flow restriction, BFR, low-intensity, overuse

## Abstract

**Objectives:**

This study explores the feasibility and effects of low-intensity blood flow restriction exercise on forearm muscle strength and function in individuals with spinal cord injury.

**Study design:**

Pilot randomized clinical trial.

**Patients and methods:**

Ten male and female adult participants with chronic cervical and thoracic spinal cord injury underwent an 8-week low-intensity blood flow restriction exercise programme that targeted forearm muscles. Each participant’s contralateral forearm served as the control. Grip strength was the primary outcome measure, and participants also provided qualitative feedback on their experiences.

**Results:**

The study revealed a significant increase in participants’ forearm muscle strength on the experimental side engaged in low-intensity blood flow restriction training, with an average strength gain of 7.5 ± 0.36 kg after 16 exercise sessions (Cohen’s d = –6.32, 95% CI –8.34, –6.68). In comparison, the control side, following a conventional high- intensity exercise regimen without BFR, showed a more modest strength increase of 4.4 ± 0.67 kg. A mean Patient’s Global Impression of Change score of 2.2 reflected overall improvements in participants’ daily activities and health status.

**Conclusion:**

This study highlights the feasibility and effectiveness of low-intensity blood flow restriction exercise as a safe and promising approach to enhancing forearm muscle strength in individuals with spinal cord injury. The observed positive outcomes, coupled with a high level of participant satisfaction, underscore the potential of this innovative method to significantly improve limb muscle strength, thereby contributing to greater functional independence in this population.

## LAY ABSTRACT

Spinal cord injury often leads to severe muscle weakness and impaired function, which can significantly affect a person’s independence and overall well-being. While high-intensity resistance exercise has been used to counteract muscle weakness, it can pose a risk of overuse injuries, especially for individuals with spinal cord injury. Consequently, there is a growing interest in exploring safer and more effective rehabilitation methods. This study investigated the feasibility and effectiveness of low-intensity exercise with blood flow restriction on forearm muscle strength and function in individuals with spinal cord injury. The results showed that low-intensity blood flow restriction exercise was feasible and significantly enhanced forearm muscle strength and function in people with SCI after 16 training sessions. These findings suggest that low-intensity blood flow restriction is a valuable addition to rehabilitation programmes for individuals with spinal cord injury. It provides a safe and promising approach to muscle strengthening, which can contribute to greater functional independence in this population.

Musculoskeletal impairments have a significant impact on the quality of life of people living with spinal cord injury (SCI) (1, 2). Chronic pain, discomfort, limb muscle atrophy and weakness, and limited range of motion in the upper and lower extremities exacerbate poor health and lead to a declining quality of life (2). Although there is a wide range of physical abilities among the SCI population, for many there is excessive use and reliance on the upper limbs in performing activities of daily living (ADL). Research studies have consistently reported that individuals with SCI have lower upper limb strength and work capacity than healthy individuals (3). However, studies examining upper limb conditions in people with SCI have found that upper body exercises can increase muscle strength. This increase in strength can help individuals in wheeling motions and grasping and may even have psychological benefits (4–7). Improving upper body strength can significantly enhance an individual’s quality of life. This highlights the necessity of investigating and developing efficient exercise methodologies with the specific goal of preserving and enhancing upper limb strength and function to support ADL while prioritizing safety and efficacy with minimal associated risks. In recent years, exercising limb muscles under blood flow restriction (BFR) has been a novel technique for efficiently improving muscular strength (6, 8). To maximize muscular strength gain and induce muscle hypertrophy, current resistance exercise guidelines indicate applying loads of greater than 60% of 1 repetition maximum (RM) for a minimum of 6 weeks, 2–3 times per week, with a repetition range of 8–12 (9). When combined with BFR, resistance loads can be reduced to 20–40% 1RM, resulting in similar or better outcomes for both strength and hypertrophy (10, 11). This exercise protocol is known as low-intensity resistance training under blood flow restriction (LI-BFR). Typically, a pneumatic cuff that is connected to a personalized tourniquet system by a hose assembly is inflated at the proximal end of the limb to restrict blood flow during muscle resistance exercise.

Despite ongoing debates concerning the LI-BFR protocol, the consensus on the guidelines suggests performing 15–30 repetitions, 2–4 sets of 20–30% 1 RM, with 30-s rests in between sets, 2–3 times per week at 40–80% limb occlusion pressure (LOP) (6, 11). The 100% LOP refers to the point at which arterial blood flow to the limb is completely stopped (11). According to previous studies, 40–80% of that point is considered safe and effective for BFR use (11, 12). Previously, BFR has been studied as a clinical musculoskeletal rehabilitation tool (13).

Findings report that LI-BFR can provide an effective, safe, and tolerable resistance training approach that minimizes pain and provides comparable results to heavy-load training (11–13). Although LI-BFR training has shown significant benefits for muscle development across different populations, research on its impact, specifically on upper limb muscle development in individuals with SCI, is limited. This study aimed to explore the feasibility and effects of an 8-week protocol of LI-BFR exercise on forearm muscle strength and function in individuals with SCI. Currently, there are a few studies that have investigated the impact of LI-BFR training on skeletal muscle development in individuals with SCI (14, 15).

## MATERIALS & METHODS

### Study design

This pilot randomized clinical trial study with a control group examined the effect of an 8-week protocol of LI-BFR exercise on forearm muscle strength in participants with SCI. Each participant was assigned to both control and experimental groups, with 1 hand designated as the control and the other assigned to the LI-BFR exercise group. To minimize potential bias and confounding factors, a randomization process was employed to determine which forearm in each participant was allocated to either the control or experimental group. Randomization was performed using computer-generated random numbers and was conducted by an independent researcher not involved in recruiting or assessing participants. This study has obtained ethics approval from the University of British Columbia’s Clinical Research Ethics Board. All participants provided informed consent, and confidentiality was maintained throughout.

### Participants

Ten adult volunteers living with spinal cord injury who met specific inclusion criteria were recruited through local rehabilitation centres and online advertisements. Inclusion criteria included individuals living with complete or incomplete tetraplegia or diplegia below C1 (AIS A, B, C, D) for at least 9 months, aged between 18 and 75 years, possessing wrist extensor muscle function rated as grade 3 or 4 with minimum 45 degrees of wrist functional range of motion. Individuals with any existing injury or anatomical abnormality in the upper limbs, participants with a history of autonomic dysreflexia, and those with any contraindications to BFR exercise, such as deep vein thrombosis, cardiovascular diseases, or vascular disorders, were excluded from the study. Participants were instructed to avoid any new intensive exercises or therapies outside of their regular lifestyle during the study period. Any ongoing outpatient therapy was documented, and participants were asked to maintain their usual therapy routines without introducing any additional interventions during the study.

### Assessment procedures

Before commencing the exercise programme, all participants underwent an initial assessment session. This session involved taking baseline measurements from both the control and intervention sides. The participants’ 1 repetition maximum (1RM) for wrist-curl exercise was determined using an adjustable dumbbell. Participants started with a free weight based on their own estimate of their strength. If the initial weight was too easy, it was increased by 8 kg. If the initial weight was moderately easy, it was increased by 5 kg. If the initial weight was close to the correct difficulty, it was increased by 2 kg to determine the 1RM. Participants were given a 5-min rest period between each attempt to ensure adequate recovery and minimize fatigue. This measurement provided a reference point for individualized exercise intensities. The forearm muscle strength of the participants was also measured using a calibrated digital hand dynamometer (GRIPX, Camry, Monte, CA, USA) (16).

### Exercise protocol

The participants performed wrist-curl exercises that aimed to engage their forearm flexor and extensor muscles. Before each exercise session, a 5-min warm-up protocol was conducted using a hand-cycling device (Monark 881E, Vansbro, Sweden). The warm-up procedure was designed to prepare the participants for the exercise protocol by enhancing blood flow and gently mobilizing the wrist joints. The exercise protocol was then initiated. The control group performed forearm extensor and flexor muscle exercises using a dumbbell set at 50% of their determined 1RM without BFR. The experimental group underwent a LI-BFR exercise using a smart BFR tourniquet (Delfi PTS, Delfi Medical, Vancouver, Canada) applied to the exercising arm, restricting blood flow at 60% of their limb occlusion pressure (LOP) and using a dumbbell set at 30% of their determined 1RM. The LOP was measured automatically by the Delfi Tourniquet using its algorithm, and we were able to set the cuff pressure at 60% of LOP using the device setting. For both groups, the exercise protocol consisted of 4 sets of wrist-curl exercises for both flexor and extensor muscles. The repetition scheme included 30 repetitions (1^st^ set), 15 repetitions (2^nd^ set), 15 repetitions (3^rd^ set), and 15 repetitions (4^th^ set). Both groups engaged in the exercise intervention twice per week for a total duration of 8 weeks (16 sessions). Each session lasted roughly 17 min, 7 min for flexor sets and 7 min for extensor sets, as well as 3 min rest in between. At each session, we started with the intervention hand, followed by the control hand exercise. A wireless surface EMG sensor (FREEEMG, BTS Bioengineering, Garbagnate-Milanese, Italy) was placed on the flexor digitorum superficialis muscle to monitor forearm muscle activity during exercise. Each exercise session was supervised by trained exercise specialists to ensure proper technique and adherence to the protocol.

**Fig. 1:**
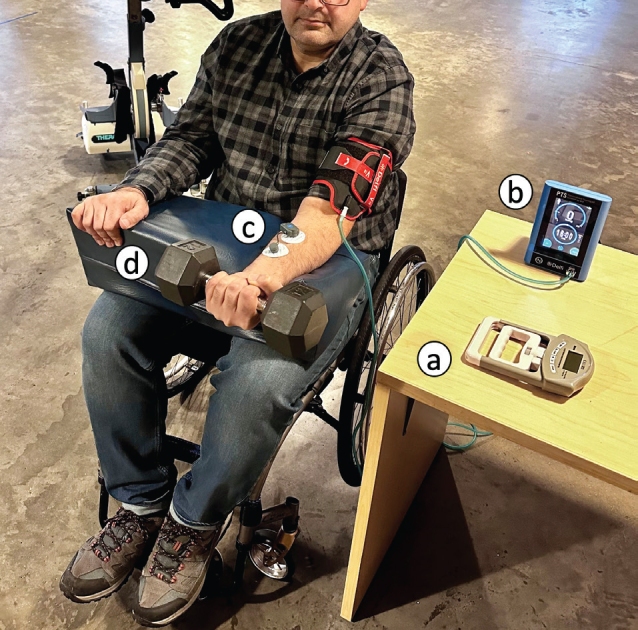
Experimental setup: (A) digital hand dynamometer, (B) personalized tourniquet system, (C) surface EMG sensor, (D) dumbbell.

During the exercise sessions, the participants’ heart rates and blood pressure were monitored in real-time using a continuous vital sign monitor (Caretaker Medical, Charlottesville, VA, USA). This monitoring method enabled the researchers to assess the participants’ physiological responses immediately. If the systolic blood pressure of any participant exceeded 20 mmHg higher than their resting blood pressure, the exercise was stopped as a safety precaution. Fortunately, we experienced no rise over the determined blood pressure limit during the 160 data collection sessions. Therefore, the exercise never needed to stop for safety purposes. Fig. 1 depicts the experimental setup.

### Outcome measures

After completing an 8-week exercise programme, bilateral grip strength was measured as the primary outcome. The efficacy of low-intensity blood flow restriction exercise was assessed qualitatively as the secondary outcome.

### Statistical analysis

The descriptive statistics were calculated to summarize the characteristics of the study variables and reported as mean (SD). A one-sample Kolmogorov–Smirnov test was used to evaluate the normality assumption. An insignificant result (*p*-value > 0.05) indicated that the data were normally distributed. Therefore, this study employed parametric tests such as paired sample *t*-tests to compare pre- and post-intervention changes in muscle strength. In addition, an independent *t*-test was utilized to compare mean differences between the control and experimental groups and investigate the significance of muscle strength improvement on average in both groups. This study also calculated and reported the effect size (Cohen’s d) to identify the magnitude of the difference between muscle strength improvement in experimental and control groups before and after the intervention. Cohen’s d was classified as small (d = 0.2), medium (0.5), and large (≥ 0.8), used to identify the standardized mean difference of the effect.

Upon completing the 8-week LI-BFR protocol, participants were required to provide detailed feedback regarding the impact of the exercise on their daily activities. They were asked to fill out the Patient’s Global Impression of Change (PGIC) scale to clearly describe any significant changes in their condition, encompassing activity levels, limitations, symptoms, emotions, and overall quality of life. The PGIC is a 7-point scale used to assess the perceived change in a participant’s health or condition following an intervention. On this scale, a rating of 1 indicates “very much improved”, while a rating of 7 corresponds to “very much worse” in terms of health status (17). Another part of the self-assessment questionnaire was a 10-point scale to examine participant satisfaction regarding the study and perceived improvements in muscle strength (17). A score of 1 indicates “not satisfied”, while a score of 10 specifies “highly satisfied”.

Furthermore, each participant was asked to rate “participants’ interest in a home-based practice of the LI-BFR to continue this exercise regularly”, with a score between 1 and 10, where 1 denotes no interest, and 10 denotes high interest. Data from these self-assessments were collected, and descriptive statistics such as frequency and percentages of the responses were conducted to evaluate the overall programme’s impact on participants’ quality of life. Data analysis has been carried out using SPSS 28.0 software (IBM Corp, Armonk, NY, USA).

## RESULTS

### Participant characteristics

Ten participants, including 8 males and 2 females living with SCI, were recruited. All participants suffered from a traumatic injury and were between 27 and 72 years old, averaging 47. 0 (SD = 14.84) years old. The mean duration of injury among participants was 145.6 months. Table I presents the demographic characteristics of the participants.

**Table I:** Participants’ characteristics and level of fitness

Participants	Fitness level	Sex	Age, years	Height (cm)	Weight (kg)	BMI	Injury level	AIS class
1	High	M	34	170	94	32	T6	B
2	Very high	M	41	177	72	23	T4	A
3	Very high	M	61	185	90	26	T4	B
4	Moderate	M	48	161	79	30	L1	B
5	High	M	52	172	78	26	T4	B
6	Moderate	M	72	176	64	20	C7	D
7	Moderate	M	60	173	72	24	T3	B
8	Mild	F	47	175	68	22	T4	A
9	Moderate	M	28	194	99	26	C7	A
10	Mild	F	27	150	59	26	L1	C
Mean			47	173	78	26		
SD			15	12	13	4		

BMI: body mass index; SD: standard deviation.

### Muscle strength

**Fig. 2:**
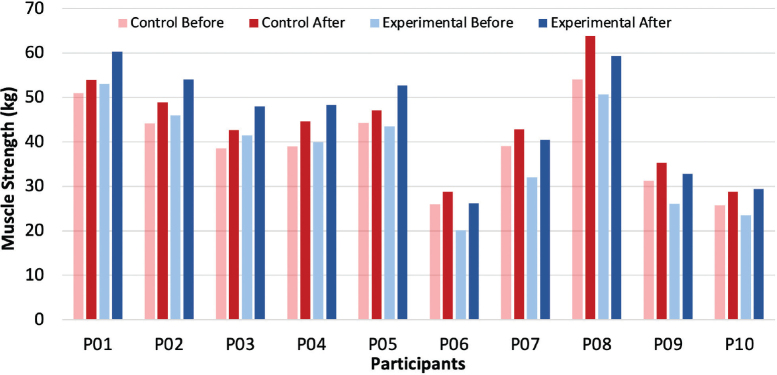
Bar charts representing the average muscle strength of (A) control and (B) experimental forearms in 10 participants before and after the 8-week exercise programme.

Collected data indicated that, on the experimental side, the mean difference in forearm muscle strength showed an increase of 7.5 ± 0.36 kg (mean ± SD) following the 16 exercise sessions. In comparison, the control side showed an increase of 4.4 ± 0.67 kg. The average muscle strength for each participant in the control and experimental groups before and after the exercise protocol is presented in Fig. 2.

### Descriptive statistics

**Fig. 3:**
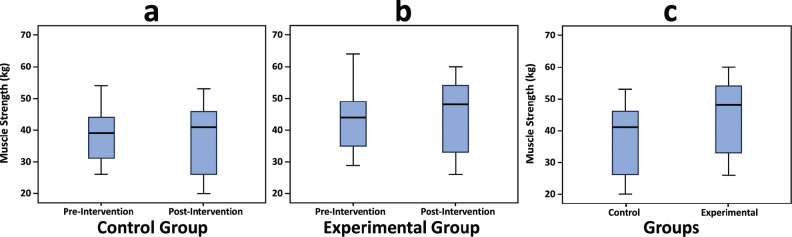
Box plot on the muscle strength changes.

The descriptive statistics of the study variables are reported in Table II. The average muscle strength after the experiment was 45.15 (12.31) kg, which was greater than before the experiment, 37.64 (11.57) kg. In addition, the average muscle strength in the control group after the intervention was 43.68 (10.89) kg, which was greater than the control group before the intervention, 39.31 (9.60) kg. This study also obtained the skewness and kurtosis values, all values were reported smaller than 1, indicating the univariate normality of the study variable. Fig. 3 demonstrates the muscle strength changes.

**Table II:** Descriptive statistics and test of normality

Factor	*n*	Min	Max	Mean (SD)	Skewness	Kurtosis	Kolmogorov–Smirnov test	
							Test statistic	*p*-value
Control before	10	26	54	39 (10)	–0.06	–0.83	0.166	0.200
Control after	10	29	64	44 (11)	0.25	0.03	0.164	0.200
Experimental before	10	20	53	38 (12)	–0.29	–1.36	0.181	0.200
Experimental after	10	26	60	45 (12)	–0.39	–1.33	0.192	0.200

### Statistical inferences

*Normality assumption.* According to the non-significant results of the Kolmogorov-Smirnov test for each group (p = 0.200 > 0.05), the normality assumption is met, indicating that all the variables are normally distributed.

*Paired* t*-test (experimental group).* The results of the paired *t*-test revealed the significant effect of the intervention in the experimental group before and after the treatment (the absolute *t* = 20 > 1.96, *p* < 0.001). In addition, the absolute effect size for the mean differences between the paired samples (before and after the intervention) in the experimental group was d = 6.32, indicating a large effect size (≥ 0.8). Individuals in the experimental group who received the exercise method showed a sixfold increase in muscle strength improvement compared with those who did not receive the exercise method (Cohen’s d = –6.32, 95% CI –8.34, –6.68) The descriptive results confirmed that the average muscle strength after the intervention (M = 45.15 kg, SD = 12.31) was higher than before the intervention (M = 37.64 kg, SD = 11.57) (Table III).

**Table III:** Results of *t*-test statistics for muscle strength improvement

Paired *t*-test: Experimental group		Mean (SD)	Paired differences				*t*	df	*p*-value	Cohen’s d
			Mean differences	Std. error difference	95% CI of the difference					
Pair 1	Before	38 (12)	–7.51	0.37	Lower	Upper	–20	9	< 0.001	–6.32
	After	45 (12)			–8.34	–6.68				
Paired *t*-test:Control group			Paired differences				*t*	df	*p*-value	Cohen’s d
			Mean differences	Std. error difference	95% CI of the difference					
Pair 2	Before	39 (10)	–4.37	0.68	Lower	Upper	–6.43	9	< 0.001	–2.03
	After	44 (11)			–5.91	–2.83				
Independent *t*-test:Post-intervention			*t*-test for equality of means				*t*	df	*p*-value	Cohen’s d
			Mean differences	Std. error difference	95% CI of the difference					
Mean differences										
Control		4.4 (2.15)			Lower	Upper	–4.07	18	< 0.001	–1.82
Experiment		7.5 (1.16)	–3.14	0.77	–4.76	–1.52				

*Paired* t*-test (control group):* The results of the paired *t*-test indicated the significant effect of the intervention before and after the treatment in the control group (the absolute *t* = 6.43 > 1.96, *p* < 0.001). In addition, the absolute effect size for the mean differences for the paired samples (before and after the intervention) in the control group was d = 2.03, indicating a large effect size (≥ 0.8). That is, in individuals who received the exercise method in the control group, the muscle strength improvement was twice as high as in individuals who did not receive the exercise method (Cohen’s d = –2.03, 95% CI –5.91, –2.83). The descriptive results indicated that the mean (SD) of muscle strength after the intervention was 43.68 (10.89) kg, which is higher than before the intervention, 39.31 (11.57) kg (Table III).

*Independent* t*-test:* The independent samples *t*-test resulted in a significant effect of the intervention between control and experimental groups (the absolute *t* = 4.07 > 1.96, *p* < 0.001). In addition, the absolute effect size for the mean differences of the 2 independent samples (between control and experimental groups) after the intervention was d = 1.82, indicating a large effect size (≥ 0.8). In individuals in the experimental group who received the exercise method, the muscle strength improvement was approximately twice as high as in individuals in the control group who received the exercise method (Cohen’s d = –1.82, 95% CI –4.76, –1.52). According to the descriptive results, in the experimental group, the mean (SD) of muscle strength was 7.5 (1.16) kg, which is higher than in the control group, 4.4 (2.15) kg (Table III).

### Survey analysis

The self-assessment data revealed that the PGIC scores were distributed as follows: 3 participants reported a rating of 1, 4 participants reported a rating of 2, 1 participant reported a rating of 3, and 2 participants reported a rating of 4. The calculated mean for these responses amounted to 2.2. According to the results of the self-assessment questionnaire, the level of satisfaction concerning muscle improvements in the hand/forearm that underwent the LI-BFR exercise method ranged from 7–10. The overall quality of life ratings after receiving the exercise protocol ranged from 2 to 9, with slight improvement to great improvement. Participants experienced slightly stronger (*n* = 5, 50%) and significantly stronger (*n* = 4, 40%) strength of the hand/forearm following the LI-BFR exercise protocol. Additionally, some 60% of the participants (*n* = 6) experienced no discomfort or pain, and some 40% (*n* = 4) experienced mild discomfort due to the exercise protocol involving BFR. The participants’ interest in home-based practice of the LI-BFR to continue this exercise regularly received a rating between 6 and 10. Most participants (*n* = 6, 60%) would definitely recommend the LI-BFR exercise to others with a similar condition, and the rest (*n* = 4, 40%) would likely recommend it to others with a similar condition.

### Participants’ feedback

After completing the study, the participants were asked to provide feedback on the exercise intervention. They were asked: “Did you notice any improvements in your daily activities or functional abilities since starting the LI-BFR exercise protocol? Please provide specific examples.” Table IV summarizes their comments.

**Table IV:** Participants’ feedback

Participant	Feedback
1	My left hand is stronger than my right hand, so I tend to use my left hand to lift heavy objects. I also feel a significant increase in my ability to wheel my wheelchair after this programme
2	This programme strengthened my left hand and made it easier for me to get out of bed and transfer to the toilet seat with no pain
3	No significant difference
4	No significant difference
5	My right grip is stronger, and its pain is reduced. No more wrist soreness or pain from prolonged wheeling or quick repetitive speed pushing for sports
6	It has improved my ability in my daily activities such as gardening, handling my cup of coffee, and swimming strokes
7	Stronger wrists, which helped me with transferring & wheeling
8	I feel stronger grasping things. Which slightly helped with transfers. Wheeling and gripping the wheel have improved. Helped me in the recovery phase of the carpal tunnel post-surgery
9	Participating in the 8-week forearm muscle exercise programme has made a big difference for me as someone with SCI. I feel much stronger in my upper body now, which means I can do things like wheeling my wheelchair and transferring more easily
10	I have noticed a huge improvement in my strength and ability to push my wheelchair. Also, my strength in grip and opening things has improved. Overall, I am so happy with the results. I definitely notice a difference in my daily life

## DISCUSSION

Our findings indicate that carrying out 16 sessions of high-intensity resistance exercise and LI-BFR exercise can significantly improve forearm muscle strength in individuals with SCI. Moreover, LI-BFR exercise provided a significantly greater positive impact on forearm muscle strength compared with high-intensity resistance exercise without BFR. Therefore, we conclude that individuals with SCI who follow an 8-week LI-BFR exercise protocol targeting their forearm muscles can achieve significant improvements in hand and forearm strength, thereby enhancing functional independence that can impact on their overall quality of life.

LI-BFR exercise involves performing muscle contractions with low intensity (20–40% of 1-repetition maximum) while partially restricting blood flow to the working muscle. This exercise has been shown to increase skeletal muscle mass and strength in healthy individuals to a similar or even greater degree than traditional high-intensity resistance training (18).

Many theories have been proposed to explain the effectiveness of BFR and shed light on its physiological mechanisms. Most researchers agree that LI-BFR leads to muscle hypertrophy through endocrine responses and muscle activation (19–21). It is commonly believed that the development of muscle strength primarily occurs through skeletal muscle hypertrophy, driven by protein synthesis. BFR has consistently demonstrated its effectiveness in triggering this physiological process (19, 20). Another explanation for the profound training effects of LI-BFR is increased muscle activations in a hypoxic intramuscular environment. When vascular occlusion is applied during high-intensity exercise, the muscle must recruit a higher number of motor units to generate the same level of force, leading to greater muscle activation (21). Greater muscle activation stimulates the Akt/mTOR pathways, increasing protein synthesis and leading to greater muscle strength (22). Evidence suggests the anaerobic environment created by vascular occlusion leads to rapid lactate accumulation, which stimulates a chemoreception pathway that regulates growth hormone secretion, an important factor in hypertrophy (21).

Muscle atrophy, impaired neuromuscular function, and varying degrees of muscle spasticity in people with SCI can make high-intensity resistance training unsafe and difficult. LI-BFR exercise could potentially be used to improve limb muscle rehabilitation and recovery in individuals with SCI. Based on current scientific research, BFR can be considered a safe intervention without any significant side effects despite reports of mild discomfort in this study. Moreover, individuals with incomplete SCI have been able to perform controlled BFR exercise safely without experiencing any additional cardiovascular stress or pain (14).

### Muscle strength improvement

As presented in Fig. 3, our results demonstrated a significant improvement in the strength of the forearm muscles in the experimental arms, emphasizing the efficacy of the LI-BFR method. While the control group exhibited expected gains from 8 weeks of traditional high-intensity resistance training, the BFR group demonstrated greater improvement than the control group. These results emphasize the potential benefits of LI-BFR in enhancing muscle strength for individuals with SCI. Our findings align with existing literature. According to a study, after only 12 LI-BFR training sessions, there was a 6.7% increase in muscle strength (18). This increase is comparable to the expected results from weeks of high-intensity training without BFR. Another study found that the leg muscles’ 1RM strength in the BFR group increased by 8.3% after 18 sessions (23). Moreover, researchers in another study observed a 16.17% increase in handgrip strength after 12 sessions of LI-BFR training (24). Handgrip strength is a major determinant of quality of life when living with SCI and may dictate the individual’s ability to conduct ADL (25). Therefore, such increases in strength are a significant contributor to a higher quality of life.

### Participant satisfaction and feedback – beyond physical improvements

Quantitative measures from the self-assessment questionnaire, including the PGIC scale and self-reported qualitative data, showed promising results after the intervention period (21, 26). In our study, 70% of participants rated their condition as “much improved” or “very much improved”, while 2 participants reported no change in health status, and none indicated a decline. Additionally, participants rated their overall satisfaction with the LI-BFR training as 6 or higher, considering factors such as convenience, effectiveness, and overall experience. These high satisfaction rates are critical as they reflect the participants’ desires and whether their expectations were met. The subjective feedback from the participants (Table IV) aligned with the quantitative data. Participants indicated significant improvements in their ADL, such as getting out of bed, transferring to and wheeling their wheelchairs, gardening, and grasping coffee cups. In general, the increase in strength of the forearm muscles appears to have a beneficial effect on the ability of participants to perform their daily tasks. The available literature also supports our view that blood flow restriction exercise positively impacts health (14, 27). A case study reported a 13.75% increase in confidence and impressive improvement in physical health and social relationships of people with SCI following LI-BFR training (27). Along the same lines, other studies emphasize improved cardiovascular health and gains in independence because of BFR (14).

### Feasibility, safety, and limitations

The BFR exercise method is new and therefore has some limitations. For instance, the cuff compression may cause discomfort. As many people with SCI struggle to maintain stable blood pressure, adding a pressurized cuff may be problematic; therefore, the method would require direct supervision from a trained individual (28, 29). While this need is acknowledged, our participants experienced no extreme pain or safety concerns during the 160 exercise sessions. The time required to set up and to clean equipment is minimal at around 5 min. However, the cost of equipment may not be feasible for all. Depending on the brand and type of BFR system, the equipment may cost $200–$4,000. The equipment used in this study lies on the latter end of the cost spectrum. While there is supporting evidence for the benefits of low-intensity blood flow restriction (LI-BFR) in improving muscle strength, further research is needed to evaluate the method’s feasibility and safety. The small sample size in our study limited our ability to account for confounding factors such as sex, age, hand dominance, and the level of injuries. This limited the generalizability of our outcomes. Additionally, the inability to blind participants, as they knew which arm had the BFR, should be addressed in future studies to minimize bias.

### Application in injury prevention and recovery

Our outcomes challenge the conventional notion of a trade-off between low-intensity training for injury prevention and high-intensity training for strength gains, suggesting that injury prevention and strength improvements need not be mutually exclusive (30, 31). Consequently, the results of this study hold significant implications for injury prevention and rehabilitation among individuals with SCI (13, 18, 31). Moreover, low-intensity training allows for a higher training frequency, unlike high-intensity training, which often necessitates reduced frequency due to greater mechanical demands and longer recovery periods (30).

### Future recommendations

Additional research is warranted to evaluate the effectiveness of LI-BFR in accelerating rehabilitation and providing opportunities for injury prevention in populations other than able-bodied individuals, including those with SCI. Further randomized controlled studies with larger sample sizes are also necessary to corroborate the results of this study. Moreover, unilateral resistance training has been shown to have a contralateral effect on non-exercising muscles (32). The impact of this should be further studied and considered in future studies.

In conclusion, the outcomes of this study confirm that low-intensity resistance training under blood flow restriction is a feasible technique that improves forearm muscle strength in individuals with spinal cord injury. These findings advocate for incorporating LI-BFR into rehabilitation programmes for SCI individuals, offering a promising and safe approach to muscle strengthening that can potentially improve their capacity for activities of daily living. The study paves the way for further research in this area, highlighting the need for more extensive studies to fully establish the benefits and scope of BFR training in the SCI population.
